# Angles-Only Navigation via Optical Satellite Measurement with Prior Altitude Constrained

**DOI:** 10.3390/s25196149

**Published:** 2025-10-04

**Authors:** Dongkai Dai, Yuanman Ni, Ying Yu, Jiaxuan Li, Shiqiao Qin

**Affiliations:** 1 College of Advanced Interdisciplinary Studies, National University of Defense Technology, Changsha 410073, China; yuying23@nudt.edu.cn (Y.Y.); lijiaxuan20@nudt.edu.cn (J.L.); 2 Nanhu Laser Labratory, National University of Defense Technology, Changsha 410073, China; 3 Department of Missile Engineering, Rocket Force University of Engineering, Xi’an 710025, China; niyuanman@163.com

**Keywords:** celestial navigation, angle-only, low-Earth orbit satellite, optical measurement

## Abstract

This paper presents an angles-only navigation (AON) method utilizing optical observations of a single satellite with known ephemeris and prior altitude constraints given by an altimeter or known topography, which can enable near-ground platforms to achieve autonomous navigation in GNSS-denied environments. By leveraging a star tracker to measure the line-of-sight (LOS) direction of a satellite against a star background, the observer’s location is resolved via triangulation under geometric constraints. Theoretical error models are derived to analyze the influence of satellite position errors, LOS direction errors, and altitude uncertainties on geolocation accuracy. Numerical simulations validate the error propagation mechanisms, demonstrating that geolocation error is primarily determined by the perpendicular projection of orbital error relative to the LOS, increases linearly with LOS distance, and is sensitive to altitude errors at low elevation angles. Ground-based experiments conducted using Globalstar satellites achieve geolocation accuracy within 250 m (RMS), consistent with theoretical predictions. The proposed method offers a practical, low-cost solution for high-precision passive navigation in maritime and terrestrial applications.

## 1. Introduction

Stars define the most fundamental and accurate inertial reference available. A celestial navigator takes advantage of stars as well-defined benchmarks and determines the attitude and geographic location of the vehicle by observing the celestial bodies [1]. The celestial navigation system (CNS) can maintain high accuracy even in a harsh electro-magnetic interference environment. It has been widely used in aircraft, ships, missiles, and other platforms as an alternative means of navigation when the global navigation satellite system (GNSS) signal is denied [2]. A traditional CNS usually uses an inertial navigation system (INS) to supply the horizontal reference and then obtains star elevation angles for navigation computation [3]. However, this horizontal reference attitude accuracy is limited by inertial sensor errors and initial misalignment. These horizontal errors are directly introduced into the celestial navigation solution and are difficult to further reduce in the absence of a GNSS signal [4].

In order to reduce the traditional celestial navigation error induced by the inertial horizontal reference, the Charles Stark Draper Laboratory proposed a concept named “Skymark”, which uses an optical tracker to sight space objects at finite distances as landmarks in the sky. Hence, the inertial navigation system is not indispensable. The feasibility of this idea is verified through simulation for the application of the ballistic missile accuracy improvement [5,6].

George H. Kaplan of the U.S. Naval Observatory presented a similar navigation method based on triangulation theory, namely, the angles-only navigation (AON) method [7,8]. It can determine the position, velocity, and orientation information for an observer using the apparent directions of close targets with known coordinates (including landmarks, satellites, etc.). According to Kaplan’s theory, the Trex Enterprises Corporation provided a design of an angles-only navigation system based on satellites measurements [9]. The test of the system was carried out, which demonstrates that the bias error of the positioning result is more than 1000 m and the peak of random error can reach 180 m [10]. This prototype system was further improved a few years later, with the expected geolocation error restricted to within 100 m [11]. The satellite ephemeris and the line-of-sight error are considered to be the main error sources of the angles-only navigation method. To restrain these errors, several research has been conducted to improve the angles-only navigation accuracy by integrating a star tracker observing satellites on a star background and an inertial measurement unit [12,13,14]. The experimental observations showed that the root mean square (RMS) of geolocation error is less than 65 m for 90% of test cases when observing GPS satellites with the aid of precise ephemerides and inertial measurement unit [15]. However, due to the low brightness of the GPS satellites, this experiment was conducted with a bulky astronomical telescope of 28 cm aperture, and it is difficult to apply it to moving platforms.

Recently, Honeywell demonstrated a celestial-aided navigation system on the E170 aircraft and achieved an accuracy of 25 m circular error with a probability of 50%. This system utilizes a star tracker to observe stars and resident space objects (RSOs) to provide a passive, non-jammable solution with GPS-like accuracy [16,17]. In addition, several military enterprises and research institutes such as Lockheed Martin, Boeing and Draper Laboratories have also published a series of patents in recent years, proposing solutions to optimize the capabilities of satellite observation and improve geolocation accuracy [18,19,20,21].

It can be seen that using an optical tracker to measure the line-of-sight (LOS) to artificial satellites with known ephemerides can effectively improve the accuracy of the celestial navigation system. It has shown the capability to navigate in an actual GPS-denied environment. Researchers have investigated navigation error of the angles-only method through simulations, but the corresponding error propagation mechanism is inadequately clarified. Moreover, these methods require the simultaneous observation of two or more satellites, or alternatively, the observation of a single satellite at different instants of time. However, astronomical observation is easy to be interfered by clouds and fog inside the atmosphere, which may degrade the visibility of the satellites [22,23]. Hence, when the celestial navigation system is employed on a near-ground platform, particularly at sea level, its data availability will be diminished. In this paper, we propose an angles-only navigation technique that relies solely on optical observations of a single satellite, coupled with known altitude information, to cater to the autonomous navigation demands of near-ground platforms. This method uses a star tracker to measure the satellite’s LOS direction relative to the stellar background, and the positions of the observed satellite can be predicted in real-time using a known ephemeris. When the initial altitude information is given by an altimeter or known topography, the navigation solution can be resolved via the triangulation theory utilizing angle-only measurement of a single satellite.

The remainder of the paper is organized as follows. In Section 2, the principle of the angles-only navigation method is depicted in detail. In Section 3, the error theory of the proposed method is established. Section 4 analyzes the position error by simulation to verify the error theory and to compare the navigation accuracy of different satellite constellations. The experimental results and the conclusion are demonstrated in Section 5 and Section 6, respectively.

## 2. Methodology

### 2.1. Angles-Only Navigation Based on Optical Observation of the Satellite

**Figure 1:**
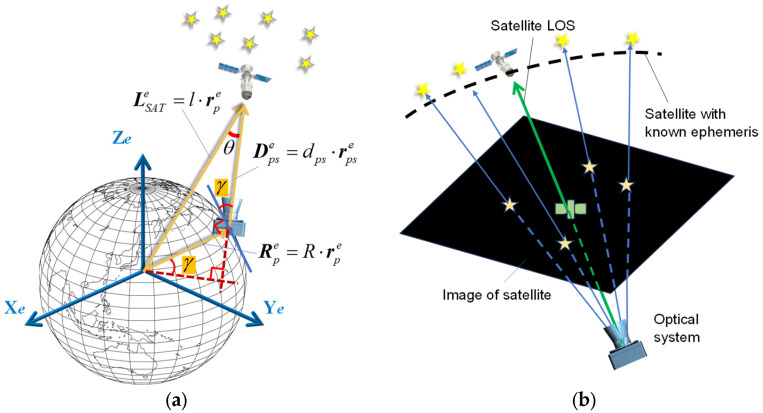
Angles-only navigation based on optical observation of the satellite: (**a**) Geometry of the satellite observation. (**b**) Determination of satellite LOS with the star field reference.

As shown in Figure 1, the angles-only navigation is realized by observing the directions of identifiable objects with known coordinates by using an optical system. The attitude of the optical system can be determined by using a set of algorithms for autonomous star tracker when more than two stars are observed [24]. Then, the line-of-sight direction of satellites can be calculated based on the pinhole model of the optical system [25].

Figure 1a presents the basic principle of satellite angle measurement-based navigation and positioning: When the camera simultaneously observes the satellite and the stars, there exists the following geometric relationship among the satellite position, the position of the observer, and the satellite LOS direction:

(1) $$d_{PS}\cdot r_{PS}^{e}=l\cdot r_{SAT}^{e}-R\cdot r_{P}^{e}$$

where the superscript *e* represents the vector in Earth-fix frame (*e*-frame), $L_{SAT}^{e}=l\cdot r_{SAT}^{e}$ are the coordinates of the satellite in *e*-frame, *l* is the distance of the satellite from the geocenter, $R_{P}^{e}=R\cdot r_{P}^{e}$ is the position of the observer, *R* is the distance of the observer from the geocenter, $D_{PS}^{e}=d_{PS}\cdot r_{PS}^{e}$ is the LOS vector of the satellite, $d_{PS}$ is the satellite LOS distance, and $r_{PS}^{e}$ is the satellite LOS direction. $r_{SAT}^{e}$, $r_{p}^{e}$ and $r_{PS}^{e}$ are all unit vectors.

Equation (1) establishes the relationship among the position of the observer, the LOS vector, and the coordinates of the satellite in the *e*-frame. In this equation, the coordinates in the *e*-frame and the LOS direction of the satellite are known, but there are still four unknown parameters, which cannot be resolved directly.

**Figure 2:**
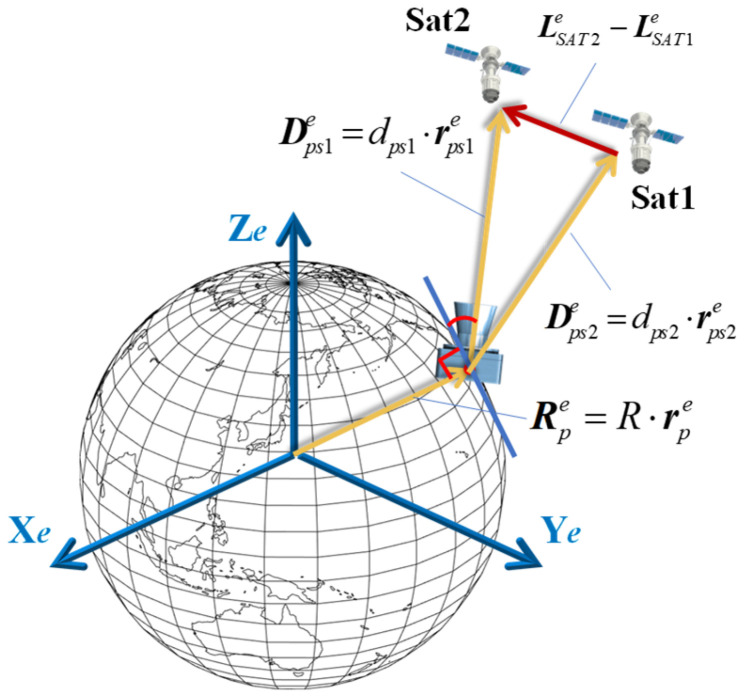
The principle of the AON method.

To solve this equation, we can observe more than two satellites for a short duration. The following section derives a general method to solve the AON problem by using observations of two satellites. As shown in Figure 2, for two satellites (Sat1 and Sat2), their coordinates in the *e*-frame can be described by vectors $L_{SAT1}^{e}$ and $L_{SAT2}^{e}$, respectively. The relative position vector between Sat1 and Sat2 can be expressed as follows:

(2) $$L_{12}^{e}=L_{SAT2}^{e}-L_{SAT1}^{e}$$

By using an optical system to observe these two satellites, the LOS vectors for each satellite can be obtained as follows:

(3) $$\left\{\begin{array}{l} D_{PS1}^{e}=d_{PS1}\cdot C_{s1}^{e}r_{PS1}^{s}\\ D_{PS2}^{e}=d_{PS2}\cdot C_{s2}^{e}r_{PS2}^{s} \end{array}\right.$$

where $r_{PS1}^{s}$ and $r_{PS2}^{s}$ are the LOS directions of satellites in the sensor’s coordinates frame (s-frame), which can be calculated with the satellites’ image pixel coordinates and the pinhole imaging model. $C_{s1}^{e}$ and $C_{s2}^{e}$ are the attitude matrix of the optical system, which can transform the vector from s-frame to *e*-frame.

The relative position vector between Sat1 and Sat2 can also be calculated using the LOS vectors of the two satellites as follows:

(4) $$L_{12}^{e}=d_{PS2}\cdot C_{s2}^{e}r_{PS2}^{s}-d_{PS1}\cdot C_{s1}^{e}r_{PS1}^{s}$$

Combining Equations (2) and (4), we obtain the following:

(5) $$L_{SAT2}^{e}-L_{SAT1}^{e}=d_{PS2}\cdot C_{s2}^{e}r_{PS2}^{s}-d_{PS1}\cdot C_{s1}^{e}r_{PS1}^{s}$$

where $L_{SAT1}^{e}$, $L_{SAT2}^{e}$, $C_{s}^{e}r_{PS1}^{s}$ and $C_{s}^{e}r_{PS2}^{s}$ can be measured or calculated directly; $d_{PS1}$ and $d_{PS2}$ are the parameters to be solved. The unique solutions for $d_{PS1}$ and $d_{PS2}$ can be obtained by solving Equation (5) using the least-squares method. Finally, the position of the observer can be directly calculated using Equation (1).

### 2.2. Simplified Angles-Only Navigation with Prior Altitude Constraint

In some applications where the altitude of the observer is known, for example, a vessel on sea level or land vehicle with known topography, the three-dimension geolocation problem can be reduced to a two-dimensional one. Utilizing the altitude constraints, the geometry of the AON method based on observing one satellite is illustrated in Figure 1a.

Using a satellite orbital prediction procedure, the satellite’s coordinates in the *e*-frame $L_{SAT}^{e}$ can be calculated in real-time. When observing the satellite using an optical system, the LOS direction in the *e*-frame $r_{PS}^{e}$ can be determined through stellar observations within the optical field of view. Given the prior altitude constraint, the distance from the observation station to the geocenter R is known. This establishes the following geometric constraint relationship:

(6) $$d_{PS}\cdot r_{PS}^{e}=L_{SAT}^{e}-R\cdot r_{P}^{e}$$

Given that the satellite’s coordinates $L_{SAT}^{e}$ and LOS direction $r_{PS}^{e}$ are known, the interior angle between these two vectors can be directly calculated as follows:

(7) $$\theta =\arccos\left(\frac{L_{SAT}^{e}\cdot r_{PS}^{e}}{l}\right)$$

In the triangle formed by the Earth’s center, satellite, and observation station, the following relationship can be established according to the cosine law:

(8) $$d_{PS}^{2}+l^{2}-R^{2}=2d_{PS}lcos\theta$$

Solving this equation, we can yield two real roots of $d_{PS}$:

(9) $$d_{PS}=lcos\theta \pm \sqrt{R^{2}-{\left(l\sin\theta \right)}^{2}}$$

Considering the geometric constraints that the observation elevation angles are greater than 0° (that is the angle formed by the LOS direction and the position vector of the observer is greater than 90°), a unique solution can be obtained:

(10) $$d_{PS}=lcos\theta -\sqrt{R^{2}-{\left(l\sin\theta \right)}^{2}}$$

In the *e*-frame, the vector from the Earth’s center to the observation point can be expressed as follows:

(11) $$R_{P}^{e}=R\cdot r_{P}^{e}=L_{SAT}^{e}-d_{PS}r_{PS}^{e}$$

The above method can determine the position of the observer through a single observation of one satellite when the altitude of observation station is known.

## 3. Positioning Error Theory of the AON Method

According to the AON theory described in Section 2.2, the observer’s position vector is determined using the satellite’s coordinates in the *e*-frame, the LOS direction from the observer to the satellite (in the *e*-frame), and the known distance from the observer to the geocenter. The geolocation errors primarily originate from measurement inaccuracies of these parameters. To reveal the mechanisms behind positioning errors induced by various factors, we will derive analytical expressions for the geolocation errors based on the fundamental principles of the AON method.

### 3.1. Satellite Ephemerides Error

According to satellite orbital dynamics theory, the positions of satellites can be predicted in real time with publicly available ephemerides information. However, due to measurement errors and inherent inaccuracies in the theoretical models, the ephemerides may induce satellites position errors ranging from hundreds of meters to a few kilometers after several hours of propagation for low-Earth orbit (LEO) satellite constellations, such as Starlink.

Considering the satellite position error $\delta L_{SAT}^{e}$, the geolocation Equation can be rewritten as follows:

(12) $$R\cdot {\tilde{r}}_{P}^{e}=L_{SAT}^{e}+\delta L_{SAT}^{e}-{\tilde{d}}_{PS}\cdot r_{PS}^{e}$$

where ${\tilde{r}}_{P}^{e}=r_{P}^{e}+\delta r_{P}^{e}$, ${\tilde{d}}_{PS}=d_{PS}+\delta d_{PS}$. $\delta r_{P}^{e}$ and $\delta d_{PS}$ represent the geolocation error of the observation station and the LOS distance error, respectively. The geolocation error can be expressed as follows:

(13) $$R\cdot \delta r_{P}^{e}=\delta L_{SAT}^{e}-\delta d_{PS}\cdot r_{PS}^{e}$$

Case A: Satellite’s position error is parallel to the LOS direction

When $\delta L_{SAT}^{e}$ is parallel to the LOS direction, we have the following:

(14) $$\delta L_{SAT}^{e}=\delta l\cdot r_{PS}^{e}$$

Under this condition, Equation (12) can be expressed as follows:

(15) $$R\cdot {\tilde{r}}_{P}^{e}=L_{SAT}^{e}-\left(d_{PS}+\delta d_{PS}-\delta l\right)\cdot r_{PS}^{e}$$

According to the law of cosines, we obtain the following:

(16) $$R^{2}=l^{2}+{\left(d_{PS}+\delta d_{PS}-\delta l\right)}^{2}-2l\left(d_{PS}+\delta d_{PS}-\delta l\right)\cos\theta$$

Expanding Equation (16) and simplifying it using Equation (8) yields the following:

(17) $$\left(\delta d_{PS}-\delta l\right)\left(2d_{PS}-2l\cos\theta +\delta d_{PS}-\delta l\right)=0$$

To ensure that the above equation holds universally, the following condition must be satisfied: $\delta l=\delta d_{PS}$.

According to Equation (13), we obtain ${\tilde{r}}_{P}^{e}=r_{P}^{e}$. It can be seen that satellite orbit prediction errors parallel to the LOS direction do not introduce geolocation errors, that is,

(18) $$\delta R_{P}^{e}=0$$

Case B: Satellite position error is perpendicular to the LOS direction

When the satellite position error is perpendicular to the LOS direction, we have $\delta L_{SAT}^{e}=\delta l\cdot r_{PS⊥}^{e}$. Substituting it into Equation (12), the following relationship is obtained:

(19) $$R\cdot {\tilde{r}}_{P}^{e}=L_{SAT}^{e}+\delta l\cdot r_{PS⊥}^{e}-{\tilde{d}}_{PS}\cdot r_{PS}^{e}$$

Taking the norm of both sides of Equation (19) yields the following:

(20) $$R^{2}=l^{2}+\delta l^{2}+{\left(d_{PS}+\delta d_{PS}\right)}^{2}+2l\sin\theta \cdot \delta l-2l\cdot \left(d_{PS}+\delta d_{PS}\right)\cos\theta$$

Substituting Equation (8) into Equation (20) and rearranging the equation yields the following:

(21) $$\delta {d_{PS}}^{2}-2\left(l\cos\theta -d_{PS}\right)\cdot \delta d_{PS}+\delta l^{2}+2l\sin\theta \cdot \delta l=0$$

Using the geometric relationship $l\cos\theta -d_{PS}=R\sin\gamma$ and lsinθ=Rcosγ, the root of Equation (21) can be determined as follows:

(22) $$\begin{array}{cc} \delta d_{PS} & =R\sin\gamma -\sqrt{R^{2}\sin^{2}\gamma -2\delta l\cdot R\cos\gamma -\delta l^{2}}\\ & =R\sin\gamma -\sqrt{R^{2}-{\left(R\cos\gamma +\delta l\right)}^{2}} \end{array}$$

Based on Equation (17), the geolocation error can be obtained as follows:

(23) $$\delta R_{P}^{e}=\left(\delta l\cdot {r_{PS}^{e}}^{⊥}-\delta d_{PS}\cdot r_{PS}^{e}\right)$$

### 3.2. Satellite LOS Direction Error

The satellite LOS direction can be calculated using the attitude of the optical system and the satellite’s image coordinates:

(24) $$r_{PS}^{e}=C_{s}^{e}r_{PS}^{s}$$

where $C_{s}^{e}$ is the attitude matrix of the optical system with respect to the *e*-frame; the LOS direction $r_{PS}^{s}$ can be calculated using the satellite’s image coordinates $\left(u,v\right)$ and the focal length of the optical system f:

(25) $$r_{PS}^{s}=\frac{1}{\sqrt{u^{2}+v^{2}+f^{2}}}{\left[\begin{array}{ccc} u & v & f \end{array}\right]}^{T}$$

Considering the camera attitude error $\varphi^{e}$ and the satellite image coordinates error $\left(\delta u,\;\delta v\right)$, the total LOS direction error can be obtained by the following:

(26) $$\delta r_{PS}^{e}\approx \left[\varphi^{e}\times \right]r_{PS}^{e}+C_{s}^{e}\delta r_{PS}^{s}$$

where $\left[\varphi^{e}\times \right]$ is the skew-symmetric matrix representation of the attitude error. For most optical systems, the focal length *f* is significantly larger than the satellite’s image coordinates *u* and *v*; thus, the higher-order terms can be neglected, leading to $\delta r_{PS}^{s}\approx {\left[\begin{array}{ccc} \delta u & \delta v & 0 \end{array}\right]}^{T}/f$.

According to Equation (11), LOS direction errors induce geolocation errors as follows:

(27) $$\delta R_{P}^{e}=-{\tilde{d}}_{PS}\left(\left[\varphi^{e}\times \right]r_{PS}^{e}+C_{s}^{e}\delta r_{PS}^{s}\right)$$

### 3.3. The Error of Geocentric Radius of the Observer

When the observer is at sea level, variations in the Earth’s radius at different latitudes introduce errors in the Earth’s radius calculation based on the WGS-84 ellipsoid model, especially when the observer’s latitude is unknown. If the observer is not at sea level, terrain undulations will also lead to errors in the prior altitude. These errors both cause errors in the distance from the observer to the geocenter δR.

According to the AON method given by Equation (10), when only the altitude error of the observer exists, the parameters l and θ in the equation can be calculated using error-free measurements. To analyze the impact of δR on geolocation results, we differentiate Equation (10) and obtain the following:

(28) $$\delta d_{PS}=-\frac{1}{\sqrt{1-{\left(l\sin\theta /R\right)}^{2}}}\delta R$$

According to Equation (11), the geolocation error can be derived as follows:

(29) $$\delta R_{P}^{e}=-\frac{1}{\sqrt{1-{\left(l\sin\theta /R\right)}^{2}}}\delta R\cdot r_{PS}^{e}$$

## 4. Numerical Simulation and Error Analysis

This section validates and analyzes the error theory of the AON geolocation method through numerical simulations. The impacts of satellite orbit prediction errors, satellite LOS direction errors, and altitude errors on geolocation results are individually examined.

### 4.1. Geolocation Error Induced by Satellite Position Error

In the simulation, the observer station is located at a fixed point (E112.99°, N28.22°, 0 m altitude), and the orbits of observed satellites are at an altitude of 500 km. Different satellite LOS elevation angles are configured, and error-free satellite positions are generated corresponding to their LOS direction. Subsequently, varying levels of satellite orbit prediction errors are introduced to the satellite position.

**Figure 3:**
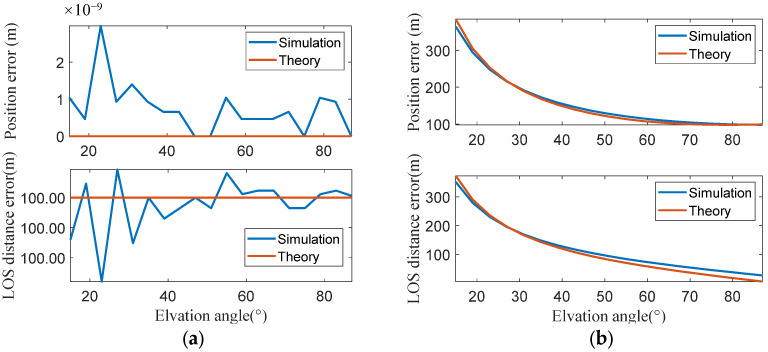
Geolocation error induced by satellite position error: (**a**) Geolocation errors and LOS distance errors are not affected by the elevation angles when the satellite orbit prediction errors are parallel to the LOS direction. (**b**) Geolocation errors and LOS distance errors decrease as the elevation angles increase when the satellite orbit prediction errors are perpendicular to the LOS direction.

According to the error theory established in Section 3, the components of satellite orbit prediction errors perpendicular and parallel to the LOS direction exhibit distinct error propagation characteristics on geolocation results. The simulation introduced 100 m satellite orbital prediction errors in both directions parallel and perpendicular to the LOS, respectively. The observer’s position is solved with the AON method, and the geolocation errors are analyzed. The simulation and theoretical geolocation errors are shown in Figure 3. When the satellite coordinate error is parallel to the LOS direction, it can be seen that the satellite LOS distance calculation error equals the orbit prediction error, and the geolocation error remains zero under different observation conditions. When the satellite coordinates error is perpendicular to the LOS direction, both the LOS distance error and the geolocation error decrease as the satellite elevation angle increases. When the satellite elevation angle reaches 90°, the geolocation error $\delta d_{PS}$ equals the orbit prediction error δl. The comparison between simulation results and theoretical results given by Equations (19) and (23) show minimal deviation.

**Figure 4:**
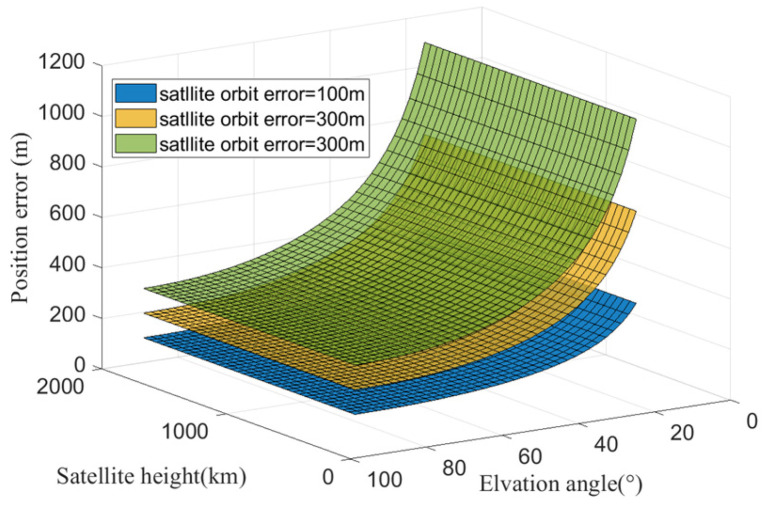
Positioning errors caused by satellite orbit errors perpendicular to the LOS direction decrease as satellite elevation angles increase, and they remain constant for different orbital heights.

To further compare and analyze the impact of orbit prediction errors on geolocation accuracy when observing satellite constellations at different orbital heights, the simulation was conducted with satellite orbit prediction errors (perpendicular to the LOS direction) with magnitudes of 100 m, 200 m, and 300 m, while varying the satellite orbital height (ranging from 300 km to 2000 km) and satellite elevation angle (ranging from 15° to 90°). The geolocation errors are shown in Figure 4. It can be seen that for a given satellite orbit prediction error, the induced geolocation error is independent of the satellite orbital height but is affected by the observation elevation angle. As the satellite orbit prediction error increases, the AON geolocation error exhibits linear growth.

Based on the comprehensive simulation results, it can be concluded that the geolocation errors caused by satellite orbit prediction errors are solely related to their projection in the direction perpendicular to the LOS, and they are independent of the satellite orbital height. This conclusion is consistent with the error theory presented in Section 3.

### 4.2. Positioning Error Induced by LOS Error

The satellite LOS direction error is caused by attitude errors of the optical system and satellite image coordinate errors. To simplify the simulation analysis, a fixed angular deviation is introduced to the LOS direction to generate LOS measurement values, which are then used for AON geolocation computation.

**Figure 5:**
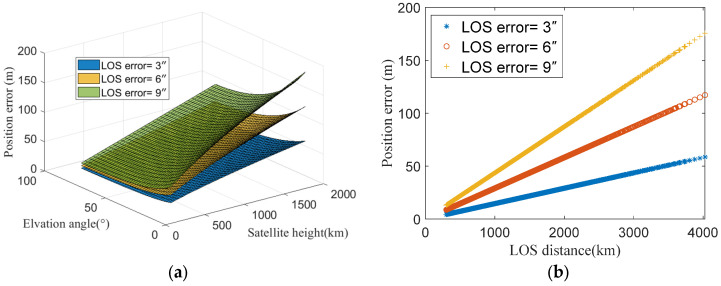
Positioning errors induced by LOS errors: (**a**) Positioning errors caused by LOS direction error vary with satellite elevation angle and orbital height. (**b**) Positioning errors are directly proportional to the LOS distance with different LOS direction errors.

The simulation was configured with satellite LOS direction errors set at 3″, 6″, and 9″, while varying the satellite orbital height (ranging from 300 km to 2000 km) and observation elevation angle (ranging from 15° to 90°). The geolocation errors are shown in Figure 5. The results demonstrate that for a given LOS direction error, the geolocation error increases linearly with satellite orbital height while decreasing with higher observation elevation angles.

From the geolocation error mechanism described in Equation (28), geolocation errors induced by LOS direction error are proportional to the LOS distance. Increasing orbital height or decreasing elevation angle both lead to greater LOS distances. Figure 5b presents the geolocation errors corresponding to the LOS distances. It reveals a clear linear relationship between the geolocation error and LOS distances. Moreover, as the LOS direction error increases, the geolocation error also exhibits linear growth. The simulation results are consistent with the theoretical analysis.

### 4.3. Positioning Error Induced by Altitude Error of Observer

The proposed AON method utilizes the Earth’s radius as a constraint for geolocation. Since the Earth’s radius varies with latitude and longitude according to the WGS-85 model, when the observer’s location is unknown or contains a large error, the calculated Earth’s radius will also exhibit corresponding altitude errors.

**Figure 6:**
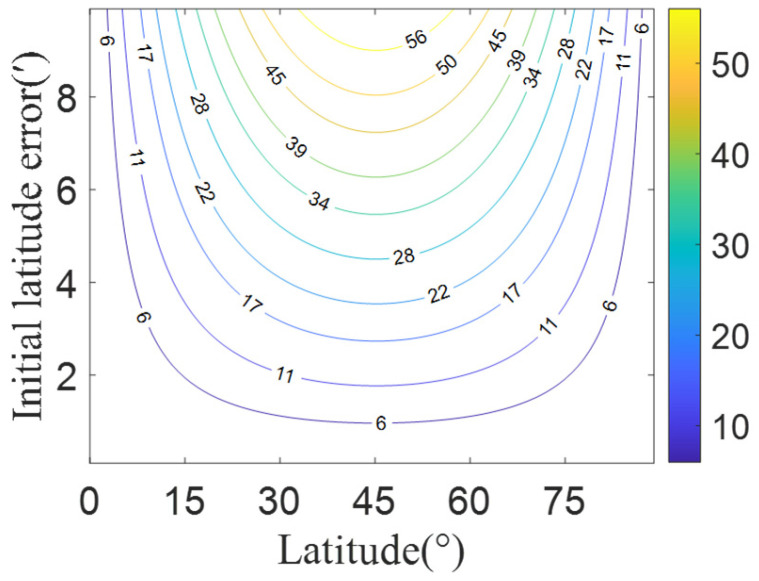
Earth radius calculation error introduced by the position error of the observer.

Figure 6 presents the maximum calculation errors of Earth’s radius under different latitude conditions (ranging from N0° to N90°) when varying latitude errors of the observer (ranging from 1′ to 10′). It can be seen that as the initial latitude error increases, the Earth’s radius calculation error shows an increasing trend. For a fixed latitude error, the maximum Earth’s radius calculation error occurs at 45° latitude, where a 2′ initial latitude error will induce approximately 10 m of Earth’s radius calculation error. The Earth radius error directly results in geocentric distance errors.

**Figure 7:**
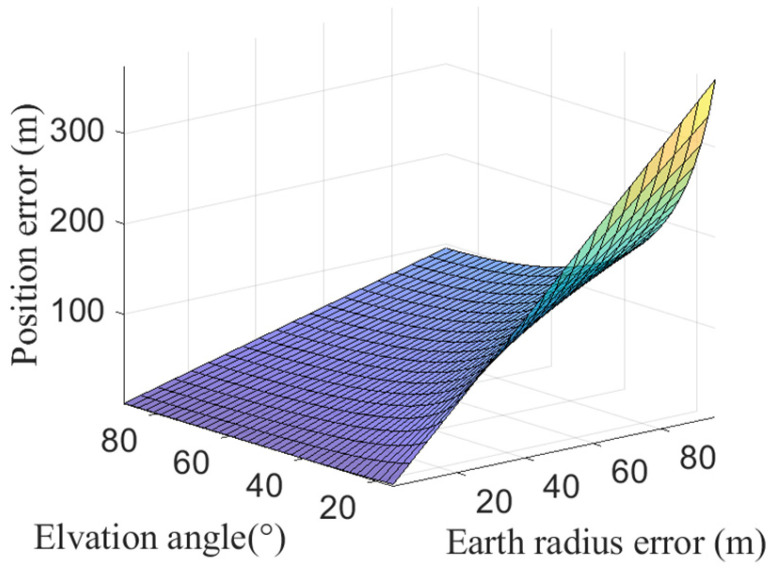
Positioning errors caused by δR increase as satellite elevation angles decrease.

To further investigate the influence of δR on geolocation accuracy, simulations were conducted with a constant satellite orbital height of 500 km, while varying both the satellite observation elevation angle (15°~90°) and δR (10 m~100 m). The corresponding geolocation errors are presented in Figure 7. It can be seen that geolocation errors are directly proportional to δR for a given observation elevation angle. Additionally, the geolocation errors induced by δR exhibit an accelerating growth trend as the satellite observation elevation angle decreases.

## 5. Experimental Results and Discussion

This section validates the performance of the AON method through nighttime ground-based satellite observation experiments and analyze the impacts of satellite position errors, LOS direction errors, and altitude errors on geolocation accuracy.

### 5.1. Experimental Setup

With the deployment of LEO satellites in recent years, several LEO satellite mega constellations, such as Starlink, OneWeb, and Globalstar, have become operational and can be observed worldwide [26]. We conducted ground-based experiment to observe the Starlink and Globalstar satellites and verified the positioning accuracy of the AON algorithm. The satellite parameters are presented in Table 1. It is shown that the mean orbit of the Globalstar satellite is much higher than that of Starlink, which leads to relatively lower brightness. The brightness of the satellite can also be characterized by apparent magnitude (denoted by Mv in the visual brand) and calculated by an empirical model [27]. A bigger apparent magnitude represents lower brightness.

The Simplified General Perturbations (SGP4) propagator is utilized to predict satellite orbit coordinates with the two-line mean element (TLE) sets [28,29], which are published by the U.S. Space Surveillance Network on the Space-Track website. It should be noted that the SGP4/TLE algorithm has limited accuracy. Achieving long-term precise orbit predictions is particularly challenging for LEO satellites, which can lead to significant systematic errors. The impact of these errors on positioning accuracy will be discussed in the subsequent analysis.

**Figure 8:**
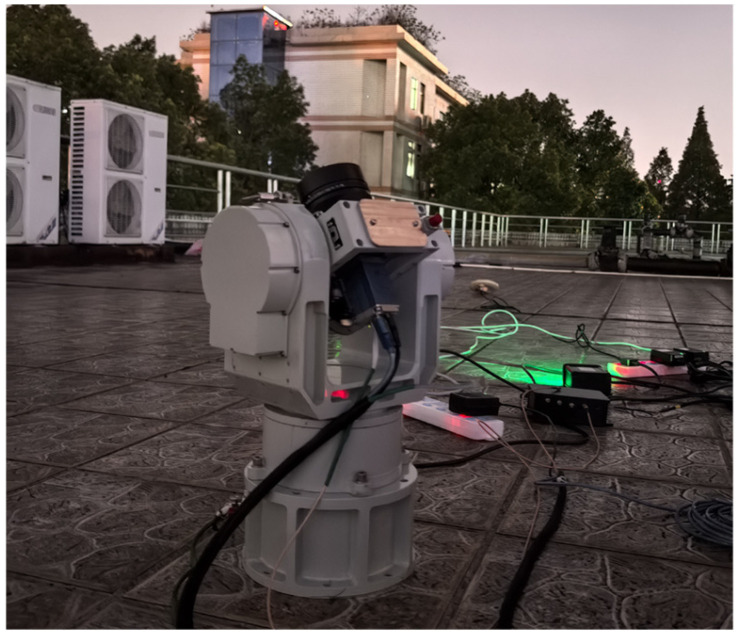
The optical system for Globalstar observation.

According to the brightness characteristics of Globalstar satellites, an optical system was constructed using a CCD camera produced by Excelitas Technologies and a 50 mm focal length commercial lens (Figure 8). The main specifications of the optical system are listed in Table 2. With a limiting detection magnitude of 7.0, this optical system is capable of observing several brighter Globalstar satellites and most of the Starlink satellites.

As shown in Figure 8, we mounted the optical system on a two-axis rotator, and deployed it in Changsha, China, located at E112.99°, N28.22°. The Globalstar and Starlink satellites were continuously tracked and imaged for positioning computation in the experiment.

**Figure 9:**
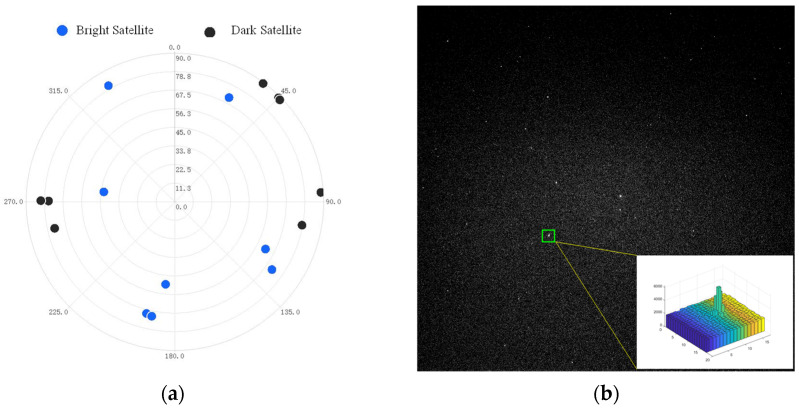
Globalstar satellites observed in the experiment: (**a**) The spatial distribution of Globalstar satellites. (**b**) A typical Globalstar satellite image.

Figure 9a shows the spatial distribution of Globalstar satellites during the observation epoch, and Figure 9b presents a typical Globalstar satellite image captured in the experiment. The brightest satellite is prioritized for observation. When the apparent magnitude of the tracked satellite exceeds Mv7.0, the optical system will automatically track the other brighter one with the aid of the two-axis rotator.

The satellite location in the image plane is extracted by a robust centroiding method and used to calculate the satellite LOS based on the pinhole model of the optical system. Then, a theoretical atmospheric refraction angle estimation model, such as the index model, Hopfield model, or Saastamoinen model, is applied to correct the satellite’s LOS direction [30]. Finally, the position of the observation station is determined by the AON method with the calculated satellite coordinates, LOS direction, and the prior altitude information given by the known topography.

### 5.2. Experimental Result

**Figure 10:**
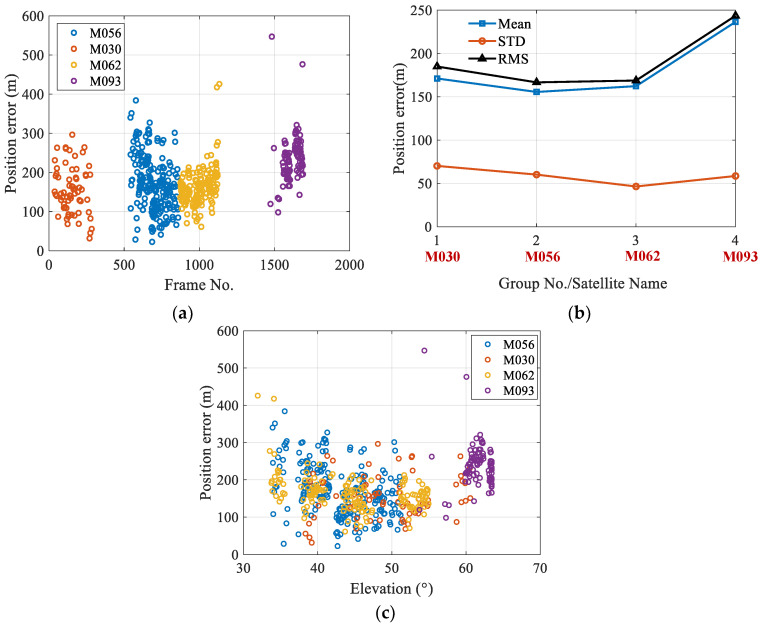
The geolocation errors for Globalstar satellite observation: (**a**) The geolocation errors via Globalstar satellite observation. (**b**) The statistical result of geolocation errors when calculated with different Globalstar satellites. (**c**) The geolocation errors with respect to elevation angles.

The experiment observed four Globalstar satellites (namely, M056, M030, M062, and M093) sequentially and calculated the observer’s position using the captured satellite images. The geolocation errors are shown in Figure 10a. It can be seen that the geolocation errors are mainly distributed between 100 m and 300 m, with the maximum error less than 600 m. Figure 10b shows the mean, standard deviation (STD) and root mean square (RMS) values of the positioning errors for each satellite. The STD errors for each satellite is less than 70 m, and the mean error is less than 250 m. These results indicate that the orbital parameters provided by Globalstar satellites have relatively high accuracy, demonstrating the strong feasibility of the AON method via Globalstar satellite observations. Satellites at low elevation angles have longer LOS distances and lower apparent magnitude, and are prone to being affected by atmospheric refraction and stray light. These factors introduce larger LOS direction errors. Figure 10c illustrates the geolocation errors corresponding to satellite elevation angles.

**Figure 11:**
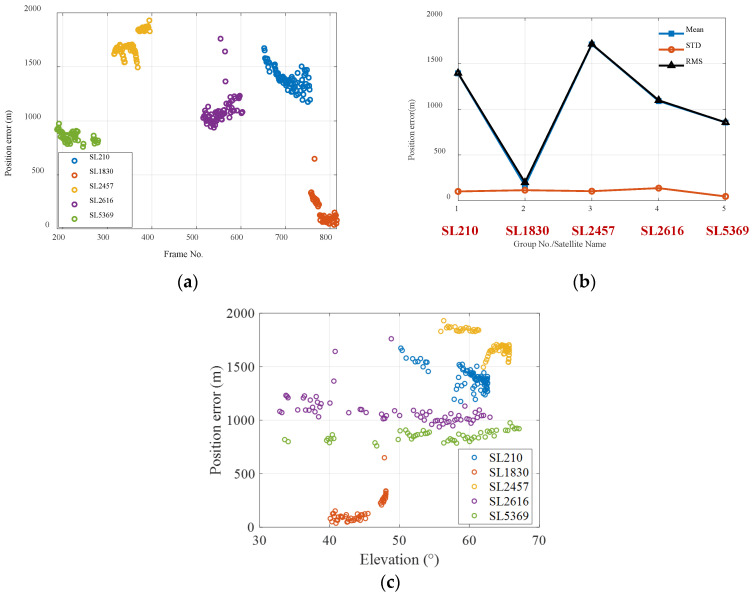
The geolocation errors for Starlink satellite observation: (**a**) The geolocation errors via Starlink satellite observation. (**b**) The statistical result of geolocation errors when calculated with different Starlink satellites. (**c**) The geolocation errors with respect to elevation angles.

Furthermore, five groups of observed Starlink satellite images (namely, SL210, SL1830, SL2457, SL2616, and SL5369) were also used for positioning calculation. The geolocation errors are shown in Figure 11a. It can be seen that the positioning error when observing the Starlink satellites is much greater than that when observing the Globalstar satellites. Figure 11b shows the mean, STD, and root mean square values of the positioning errors for each satellite. The mean position errors range from 200 m to 1700 m, and the STD errors remain about 100 m. Figure 11c shows the geolocation errors corresponding to elevation angles of the observed Starlink satellites.

### 5.3. Positioning Error Analysis

To experimentally validate the geolocation error theory, satellite coordinate errors, LOS direction errors, and altitude errors of the observer were introduced into the raw data, respectively. Due to the higher orbit of the Globalstar satellite, the satellite coordinates calculated by the orbital prediction procedure are more accurate. Therefore, we adopt Globalstar satellites observation data to conduct the positioning error analysis. The impacts of these error sources on positioning results were then analyzed.

**Figure 12:**
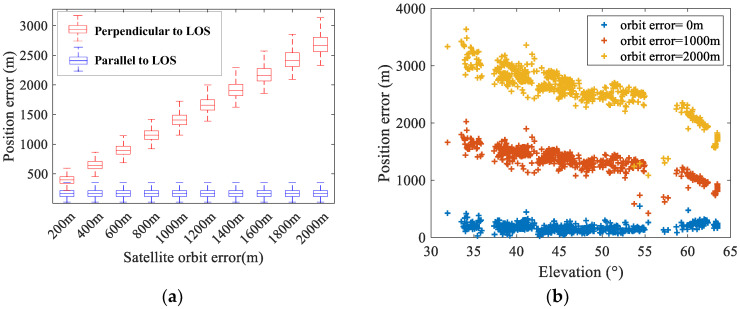
The geolocation errors induced by the satellite position errors: (**a**) The statistical results of geolocation errors in different directions. (**b**) The geolocation errors with respect to elevation angles.

Satellite coordinate errors that are perpendicular and parallel to the LOS direction are added to the original experimental data, respectively, and the positioning error statistics are shown in Figure 12a. It can be seen that satellite position errors parallel to the LOS direction do not affect geolocation results, while errors perpendicular to the LOS direction increase the geolocation error nearly linearly; the standard deviation shows a slight increase. Figure 12b presents positioning errors corresponding to satellite elevation angles when different magnitudes of satellite position error are added. It can be observed that the geolocation errors increase significantly for satellites with lower elevation angles, consistent with simulation results.

**Figure 13:**
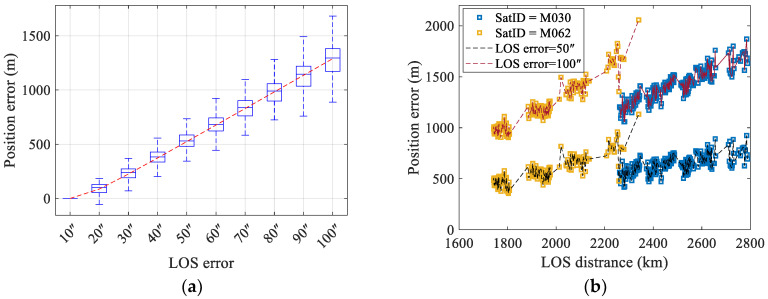
The impact of LOS direction errors on positioning results: (**a**) The statistical result of geolocation errors induced by LOS direction errors. (**b**) Geolocation errors induced by the LOS direction with respect to elevation angles.

A group of satellite observation data with higher SNR (satellite ID is M056) was selected for AON positioning calculation, and LOS direction errors were added to the original data. When different magnitudes of LOS direction errors (10″~100″) were introduced, the statistical results of geolocation errors are shown in Figure 13a. It can be observed that the geolocation errors increase approximately linearly with the growth of LOS direction errors. Figure 13b presents the geolocation errors corresponding to LOS distance when LOS direction errors are set to 50″ and 100″, respectively. The results show that the geolocation errors grow approximately linearly with increasing LOS distance, and a larger LOS direction error accelerates the growth trend of the geolocation errors.

**Figure 14:**
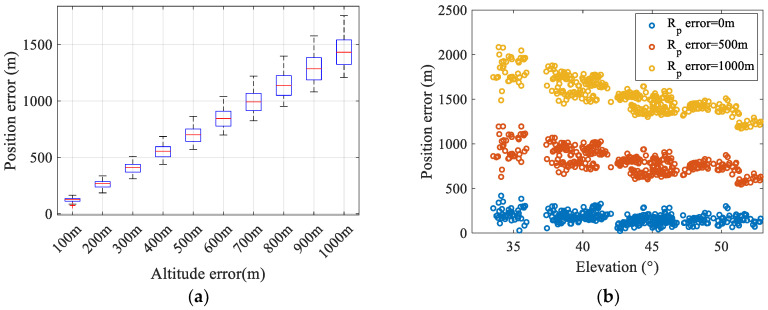
The impact of altitude errors on geolocation results: (**a**) The statistical result of geolocation errors induced by altitude errors. (**b**) The impact of altitude errors on geolocation errors with respect to elevation angles.

According to the simulation results in Section 4, the Earth’s radius calculation error caused by observer position is relatively small. However, when the observation station is located on land, terrain variations introduce significant altitude errors, which substantially impact geolocation results. Figure 14a shows the statistical results of geolocation errors when different altitude errors are applied. It can be seen that the mean value of the geolocation error increases linearly with altitude error, and the STD of errors also increases significantly. Moreover, the geolocation errors caused by altitude errors are more significant when observing satellites at lower elevation angles (as shown in Figure 14b).

In the ground-based satellite observation experiments, the TLE orbital parameters used for satellite orbit prediction lack accuracy, introducing a fixed bias error of approximately 200 m to the satellite coordinates. This leads to a systematic geolocation error of approximately 200 m. Additionally, random errors in satellite LOS direction calculation induced by attitude determination and satellite image centroiding contribute to random perturbations in AON geolocation results (STD ≈ 50 m). Since the altitude of the observation station is precisely known in this experiment, the resulting geolocation error can be ignored. Error characteristic analysis with experimental data shows good agreement with both simulation and theoretical predictions. The primary source of geolocation error originates from inaccuracies in satellite orbit prediction. It can be estimated that the satellite coordinate errors are approximately 200–300 m based on geolocation results. When precise satellite ephemerides and a more accurate orbital dynamic model for satellite orbital prediction are applied, it is expected to significantly improve AON geolocation accuracy in the future [31].

## 6. Conclusions

This paper proposes an angles-only navigation method based on optical observation of satellites with prior altitude constraints. The proposed method overcomes the limitation of traditional astronomical navigation system, resolving the geolocation problem without the aid of an inertial navigation system and achieving high-precision geolocation through single satellite observations. It is suitable for shipborne navigation applications. Theoretical analysis and experimental verification show that the geolocation error of this method is mainly affected by satellite orbit prediction errors, and the influence of Earth radius calculation errors and satellite LOS direction errors is relatively small. The experimental results show good agreement with the theory and simulation analyses. Since the Globalstar satellites’ coordinates can be predicted by the SGP4 algorithm with relatively high accuracy, the geolocation results can reach the 200 m level. When more accurate orbit prediction models are applied, it is expected that orbit prediction errors will further reduce, thereby improving geolocation accuracy. This study provides a new approach for autonomous navigation under GNSS-denied conditions and can be integrated with autonomous navigation systems to achieve better performance. It should be noted that the positioning accuracy was only verified through static ground-based experiment. In order to apply this technology to mobile platforms, more experimental validations under dynamic conditions need to be conducted in subsequent research.

## Figures and Tables

**Table 1:** The parameters of the Globalstar and Starlink satellites.

Satellite	On-Orbit Number	Mean Orbit Height	Apparent Magnitude
Globalstar	103	1500 km	Mv6~Mv10
Starlink	>8000	550 km	Mv4.5~Mv7.2

**Table 2:** The main specifications of the optical system.

Optical Module	Parameter	Value
Camera	Resolution	1392 × 1040
	Pixel size	6.45 μm
	Quantum efficiency	65%
	Read out the noise	6~8 e^−^
	Non-uniform noise	<1%
	Exposure time	150 ms
	Spectral response	380~780 nm
Lens	Focal length	50 mm
	Optical aperture	30 mm
	Field of view	10.3° × 7.7°
	Transmittance	>0.8
	Distortion	<0.1%

## Data Availability

The original contributions presented in this study are included in the article. Further inquiries can be directed to the corresponding authors.
